# Case of Extensive Varicose Veins

**Published:** 1873-05-01

**Authors:** Daniel T. Nelson


### CASE OF EXTENSIVE VARICOSE VEINS.

BY DANIEL T. NELSON, M.D.

The following case was written out in 1865, but not published. It may be of sufficient interest to publish. It surely is an uncommon case.

Oscar W., Co. E, 13th Regiment Veteran Reserve Corps, age twenty-nine; occupation before enlistment a printer, and later a law student. When about seven years of age, while at play upon the hay in a barn, was pitched from a scaffold aud fell upon the hay below, a distance of some six or seven feet. Being unable to rise he was carried to the house. The attending physician found fractures in two places of the left femur — one just above the knee, but not complicating the joint, and the other in the middle third. The place of this last fracture is easily felt; but the other, if it ever existed, has left no relict.

At the age of sixteen years began the occupation of printer. Worked at type-setting six years, studied law three years, and has since been in the United States service.

Patient thinks the varicose veins began to appear soon after the limb was broken, probably in the summer following, the limb having been broken in the winter, though cannot fix the exact date. He remembers they were quite large and troublesome soon after he began type-setting, but they were confined to the region of the knee and below, and did not seem to increase in size or extent. In April, 1861, enlisted in a three years' regiment, but served five more before he was mustered out; was on duty all the time with his regiment, going on all the marches with it. Was in the battle of Big Bethel, and marched the day before thirty miles. In November, 1861, enlisted again in a three years' regiment (the 8th Vermont). Went with the regiment to Ship Island. One day while out on drill, after marching about in the deep sand some three or four hours, he was attacked with a severe pain in the left leg, and was obliged to fall out and go to his tent.

The next day the whole limb was much swollen and very painful, and veins very prominent. The treatment was a bandage over the whole limb, and rest. The pain and swelling soon subsided, and in a week he was on duty again.

But after this time the varicose veins were much more extensive, appearing within a few weeks in the posterior part of the thigh, the gluteal region, and in the genital organ; and since that time they seem to have slowly increased.

Patient went with his regiment to New Orleans, and did provost duty for several months, but was never able to make long marches after leaving Ship Island.

Discharged December, 1862, on account of varicose veins. Enlisted in Veteran Reserve Corps in June, 1863. In July, 1864, had an attack of hæmorrhage from the urethra. Patient thought as much as a pint passed from the urethra when it first commenced, but afterwards only a few teaspoonfuls each time after urinating. He was treated with tincture ferri chloride by the mouth, and rest in bed. In about a week he was on duty again.

Three or four weeks later he had a similar attack of bleeding, though less severe. In January a third attack occurred, much like the first. Hæmorrhage occurred every time the urine was passed; not with it, but after. It was attended with a severe smarting pain in the urethra, in the region of the bulb, and also near the end of the penis, just behind the glands.

The treatment was tannin injections, and rest in bed. Returned to duty in about ten days. The general appearance of the patient was healthy. Could walk without difficulty, certainly for short distances. On exposing the person the following extensive varicose veins were seen : beginning with the foot on the left side, all the veins seem greatly enlarged, and over the first phalanx of each toe there is a diffused varix covering the whole of the phalanx above and extending somewhat over the next. Over the metatarsal bones another varix nearly covers the dorsum of the foot, communicating freely with those of the toes. Directly in front of the articulation of the tibia with the astragalus is a varix of the size of an English walnut, not as diffused as the others. On the plantor surface the same tendency to varix is seen, though there are no larger ones. Along the calf of the leg the internal and, to some extent external, saphenous vein is enlarged. Anteriorly the superficial veins are little enlarged, and posteriorly not very much. But at the knee the external saphenous forms a varix three inches in length by two-thirds of an inch in width. Above the knee the internal saphenous is not varicose. In the muscles on the posterior aspect of the thigh the deep veins are much enlarged, as are also the superficial ones; but in the anterior two-thirds, including the region of the great vessels, the veins seem to be perfectly normal. In the glutial region the lower two-thirds of these muscles are filled with enlarged veins and varices; and some are so superficial it would seem there was danger of rupture externally. The smaller varices are from the size of a pea to that of a filbert, while deep in the muscles a diffused varix some three inches in diameter can be felt.

But the most remarkable of all are the genital organs. The penis is so filled with venous blood that it looks gangrenous. At the root of the penis are several projecting bodies of the size and shape of a large nipple, having the same dark venous color. These seem to have been originally varices, but the blood has undergone some changes, as they have not the feel of blood, though they retain its color.

Between the anus and scrotum, and extending forward along the urethra so as to include the bulb, is a varix of the size of a hen's egg. But there is no appearance of hæmorrhoids, and the patient never has complained of piles. The scrotum is about the normal size, but the superficial veins are greatly enlarged and contorted; but the veins of the cord do not seem to be diseased in the least; no varicocele whatever can be felt.

Along the body of the penis all the veins are greatly enlarged, but so regularly that they give the organ the peculiar blue appearance described; but there are no well marked varices except in the corpus spungiosa, near the fossa naviculosis, where is one about the size and shape of a tamarind seed.

And, indeed, the gland is little else than a diffused varix, so completely is it injected with venous blood, particularly when allowed to hang in a dependent position for a time, so as to allow the blood to gravitate. Indeed the organ may be said to be subject to a two-fold erection — an active or normal, and a passive or venous — when allowed to hang in a dependent position. When passively erected it is larger, softer, and darker in color than when actively erected.

The gland, when passively erected, is about twice the usual size of a gland normally erected, and the whole organ is proportionably enlarged. At the base of the corona glandis the follicles of Tyson are loaded with their peculiar secretion, and being for some reason unable to discharge their contents, they appear as numerous white specks in the mucous membrane of the size of millet seeds. Many of the sebaceous glands in the skin of the penis are filled with their secretion, presenting similar whitish spots. Beside these there are numerous red specks about the size of a pin head, which seem to be of the nature of superficial varices, only filled with red blood. Altogether it is a remarkably large and variegated organ.

To review in brief the points of interest, seem to be the little trouble to the patient which so extensive varicose veins have caused; the position of the enlarged veins in the *posterior third* only of the thigh; their extent in the genitals and vicinity, uncomplicated with varicocele or piles; the enlarged and variegated penis.

Was the fracture of the femur in childhood in any way the cause of the enlarged veins?

---

Dr. Abner Phelps died in Boston February 24th, aged 95 years. In 1826, when a member of the Legislature, he proposed the first railroad bill ever drafted in America.

